# Pediatric tuina for the treatment of fever in children: A protocol for systematic review and meta-analysis

**DOI:** 10.1097/MD.0000000000021664

**Published:** 2020-08-14

**Authors:** Long-Fang Chen, Ming Yin, Xing Dong, Jia-Xi Zou, Bai-Xue Wang, Ji Chen

**Affiliations:** a School of Basic Medicine; b School of Foreign Languages, Chengdu University of Traditional Chinese Medicine, Chengdu, Sichuan, China.

**Keywords:** children, fever, meta-analysis, pediatric tuina, protocol, systematic review

## Abstract

**Background:**

Infantile fever is a common symptom of the pediatric diseases, which is often caused by cold, food accumulation, or other pathogenic factors. Pediatric tuina is regarded as an acceptable non-pharmaceutical therapy for children with optimal effects, which has been widely used for infantile fever around China. But there is still a lack of systematic evaluation and research on its safety and effectiveness during the treatment of infantile fever. Thus the protocol is to collect clinical evidence and demonstrate the efficacy and safety of antipyretic manipulation by pediatric tuina.

**Methods:**

The systematic electronic search will be executed in Cochrane Library (1991–2020.6), EMBASE (1980–2020.6), PubMed (1996–2020.6), WHOICTRP (2004–2020.6), Web of Science (1900–2020.6), CNKI (1994–2020.6), CBM (1994–2020.6), WANFANG (1980–2020.6), and VIP (2000–2020.6) Database. The Review Manager (V.5.3) will be use to assess the risk of bias and data analyses. The methodological quality will be assessed by using the online GRADEpro tool. If the quality of numeric data is favorable, a meta-analysis will be carried out.

**Results:**

A high-quality evidence of pediatric tuina for the treatment of infantile fever.

**Conclusion:**

The systematic review will provide a reliable basis for judging whether pediatric tuina is safe and effective in the treatment of pediatric fever.

**INPLASY Registration number:**

INPLASY202060032

## 1 Introduction

Infantile fever is a common symptom of the pediatric diseases, accounting for 30% of pediatric emergencies.^[1]^ It is often caused by upper respiratory tract infection, pneumonia, diarrhea, indigestion, and other factors. It is generally believed that fever is a defense response by the body to fight against and eliminate germs. However, long-lasting high fever can lead to abnormal regulation of various organs and tissues in children's body, even seriously lead to convulsions, destroy brain tissue, leave irreversible sequelae, and bring harm to children's health.^[2–4]^ Western medicine believes that fever is caused by a series of reactions caused by internal antigen-antibody complexes or external bacteria, viruses, fungi, spirochetes, etc. Therefore, oral ibuprofen and acetaminophen are often given in clinic. It has a certain curative effect, but the side effects are unavoidable.^[5,6]^ So parents are looking for greener antipyretic methods.

Pediatric tuina is regarded as an acceptable non-pharmacotherapy for children with positive effects on pediatric disease, which has been widely used in China.^[7–9]^ Pediatric tuina is a characteristic therapy in the treatment of diseases by hand manipulation, such as pushing, grasping, kneading, rubbing, and so on. Under the guidance of the theoretical basis of syndrome differentiation and treatment of traditional Chinese medicine, special parts of children's body (mostly acupoints) are selected to achieve the purpose of tonifying deficiency and purging excess, adjusting yin and yang, relaxing muscles and dredging collaterals, promoting qi, and activating blood circulation.^[10–12]^

From the clinical observation, pediatric tuina is suitable for fever caused by different reasons, which can be used alone or combined with other treatments, such as traditional Chinese medicine, acupuncture, scraping, cupping, and so on.^[13–17]^ Although pediatric tuina is popular, there is still a lack of high-quality clinical evidence to prove the safety and effectiveness of pediatric tuina in the treatment of infantile febrile disorders.

## 2 Methods

### 2.1 Selection criteria

#### 2.1.1 Types of studies

All randomized controlled trials (RCTs) that have been published and can be obtained with the languages of English and Chinese will be included, except for studies that have been published in the form of letters, reviews, abstracts or conference posters and for which full papers are not available, and the data cannot be extracted completely and duplicately.

#### 2.1.2 Types of patients

The age of participants is 14 years or under, men or women. They will comply with authoritative clinical diagnostic standards for pediatric fever, excluding those suffer from other serious illnesses, such as the diseases in heart, liver, kidney and blood system, infectious diseases, and severe hereditary diseases, without symptoms such as dizziness, convulsions, and so on.

#### 2.1.3 Types of interventions and comparisons

Children of the intervention group should receive Tuina alone or combined with integrative medicine or other conventional medicine. There would be no limitation about the type of tuina manipulation (acupoint tuina, abdominal tuina, spinal pinching), time, and treatment course of tuina. Children of the control group should receive other routine treatments, such as phototherapy, drug therapy, touch therapy, observation, and nursing.

#### 2.1.4 Types of outcomes

The significant effective rate will be used to the primary outcome, which is defined as the abatement of fever in a special period of time (meet the authoritative clinical effective standards for pediatric fever). Significant effective rate = (the number of significant effective participants/total number of participants) × 100%. Secondary outcomes will include the occurrence of adverse events.

### 2.2 Search strategy

We will search for the studies in Cochrane Library (1991–2020.6), EMBASE (1980–2020.6), PubMed (1996–2020.6), WHOICTRP (2004–2020.6), Web of Science (1900–2020.6), CNKI (1994–2020.6), CBM (1994–2020.6), WANFANG (1980–2020.6), and VIP (2000–2020.6) Database, the language of studies will be limited in English and Chinese. This protocol was registered with the “International Platform of Registered Systematic Review and Meta-Analysis Protocols” (INPLASY), registration number is INPLASY202060032. Because an evidence-based medical research will be performed, so there is not necessary for the ethical approval. The search strategy is described in Table 1.

**Table 1:**
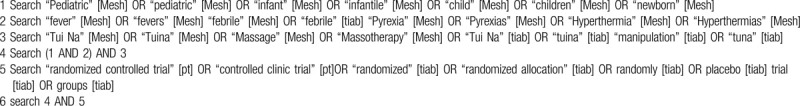
Search strategy of PubMed.

### 2.3 Data collection and analysis

#### 2.3.1 Selection of studies

**Figure 1:**
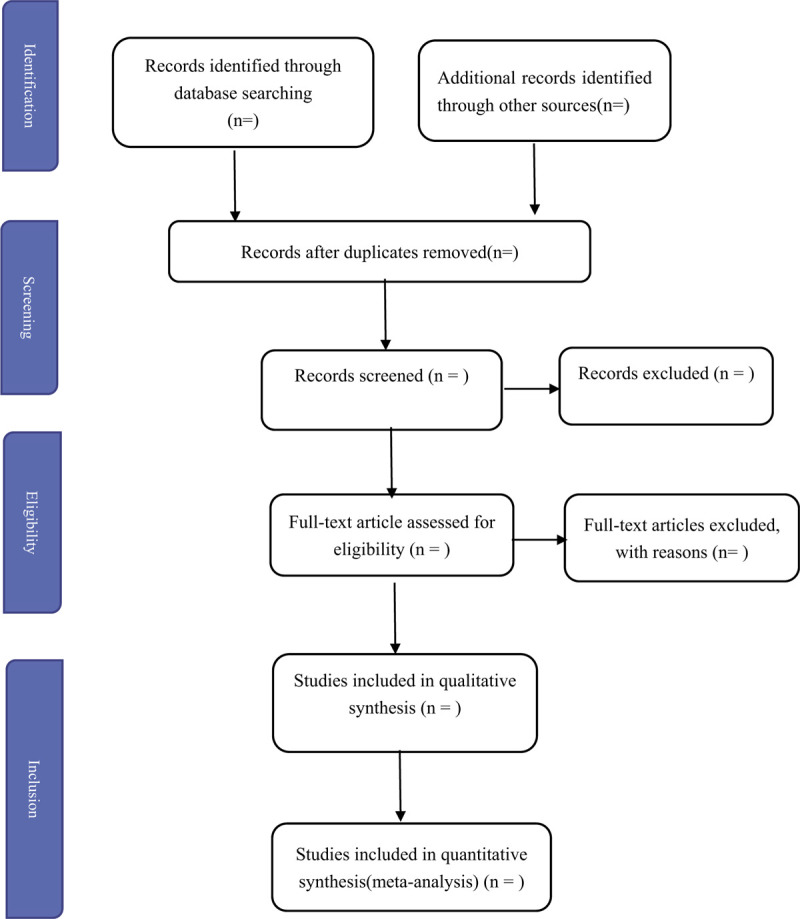
The PRISMA flow diagram of the study selection process. PRISMA = preferred reporting items for systematic reviews and meta-analysis protocols.

Two reviewers (DX and YM) will search literatures independently through strategy above and fill the diagram (Fig. 1). The literatures are selecting by two methods: reading the title and abstracts; reading the full-studies. Then they will import all selected studies into Endnote and deleted the duplicates. If the data are unreported, they will try to contact the authors to request the original data, when those are necessary for the completion of the systematic review. What's more, the studies which are published as a letter, reviews, abstract, or conference poster will be excluded unless sufficient data can be acquired from the authors.

#### 2.3.2 Data extraction

We will use WPS Office (V 11.1) to design a standard extraction form, which will contain all the necessary information from the selected studies. Data items to be extracted will include study characteristics (publication year, name of the first author, total sample size, and fever duration), patients (course, sex, age, number), intervention and control (method of intervention, method of control, sample size, and treatment time) and outcomes (clinical efficacy, adverse reactions), and so on. Two reviewers (W-BX and DX) will independently use it to extract data. Disparities will be resolved by discussion or referred to a third reviewer (Z-JX).

#### 2.3.3 Assessment of risk of bias in included studies

Two reviewers (Z-JX and DX) will use the Cochrane risk-of-bias tool to evaluate the included RCTs’ risk of bias, in terms of selection bias (random sequence generation, allocation concealment), performance bias (blinding of participants and personnel, blinding of outcome assessment), attrition bias (incomplete outcome data), reporting bias (selective reporting), and other bias. “high risk,” “low risk,” or “unclear risk” will be used to determine the result above. Disparities will be resolved by discussion and consultation with other authors in our group, then made a judgment basing on consensus.

#### 2.3.4 Measures of treatment effect

The dichotomous outcomes of participants experiencing in the studies will be recorded, 2 reviewers (YM and C-LF) will extract the standard deviations and means of the continuous outcomes, import the information into RevMan 5.3 (International Cochrane Collaboration Network, Nordic Cochrane Center, Oxford, UK) for data analysis. The risk ratio (RR) with 95% confidence intervals (CI) will be used to summarize the dichotomous data, and standardized mean difference (SMD) with 95% CI will be used to adopt for the continuous outcomes.

#### 2.3.5 Assessment of heterogeneity

Cochrane *Q* test is to be used to assess the existence of heterogeneity, and the extent of the heterogeneity will be quantified using the *I*^2^ statistics (large heterogeneity 50%–70%; very large heterogeneity >70%). If non-significant heterogeneity is found among pooled studies, a fixed effect model will be used to summarize the results of the studies; if the heterogeneity is found, the subgroup analysis that divide all the data into smaller units and compare them within each subgroup will be necessary.

#### 2.3.6 Assessment of reporting bias

When the amount of total included literature for data analysis is >10, a funnel plot will be conducted to assess publication bias.

#### 2.3.7 Data synthesis

According to the extraction results of clinical randomized controlled trials, if they have a similar clinical characteristics (on participants, study design, control, interventions, and outcomes) and acceptable statistical heterogeneity, a meta-analysis that pediatric tuina in the treatment of infantile fever will be carried out, or a narrative synthesis by available data will be conducted when there is a great heterogeneity within the studies (*I*^2^ > 70%).

#### 2.3.8 Subgroup analysis

If the included studies have a high heterogeneity, we will use the subgroup analysis to explore the sources of heterogeneity in the different diagnosis reasons (exogenouspathogenic facotors, indigestion, and so on), and different intervention types (only one kind of therapy or combining with other therapies).

#### 2.3.9 Sensitivity analysis

If the quality of studies is low, or the outliers that are numerically distant from the rest of the data, a sensitivity analysis will be required. We will use the iteratively removing one study at a time of ReveMan5.3 to finish the sensitivity analysis.

#### 2.3.10 Grading the quality of evidence

Quality of the included RCTs will be evaluated by GRADE judgement. The evidence quality evaluation of important results will be described in 4 levels: high, moderate, low, and very low. According to the evidence quality, the degree of recommendation will be estimated.

#### 2.3.11 Ethics and dissemination

There are no requirement of ethical approval and informed consent, because all the data generated or analyzed during this study are publicly available. The results will be published in a peer-reviewed journal or disseminated in relevant conferences.

## 3 Discussion

Infantile fever is a common symptom in pediatrics which often causes worry and stress for parents.^[18–20]^ The side effects of taking antipyretic drugs in the long-term will also have a certain impact on children's health. Pediatric tuina has a long history in China, and has remarkable curative effect in treating pediatric diseases. According to modern research, pediatric tuina cannot only reduce fever in time, but also has a sustained and stable antipyretic effect. Besides, it is a low-cost therapy which is easy to operate, and master. It can be promoted as a routine pediatric health care treatment, and parents can also learn to manipulate.^[21–23]^ This systematic review will be the first qualitative research to focus on the pediatric tuina in the treatment of infantile fever. The outcomes of this review will provide pediatricians, therapists, and parents with more information on the selection of methods in treating infantile fever, and provide a new research direction for future studies according to credibility of current evidence.

## Author contributions

**Administration:** Long-Fang Chen; Ji Chen.

**Conceptualization:** Long-Fang Chen.

**Formal analysis:** Xing Dong; Bai-Xue Wang.

**Investigation:** Long-Fang Chen; Jia-Xi Zou.

**Methodology:** Ming Yin; Xing Dong.

**Resources:** Ming Yin; Bai-Xue Wang.

**Software:** Long-Fang Chen; Ming Yin.

**Supervision:** Ji Chen.

**Visualization:** Jia-Xi Zou; Bai-Xue Wang.

**Writing – original draft:** Long-Fang Chen.

**Writing – review & editing:** Long-Fang Chen; Ji Chen.
